# Global tendencies and frontier topics in hemodynamics research of intracranial aneurysms: a bibliometric analysis from 1999 to 2022

**DOI:** 10.3389/fphys.2023.1157787

**Published:** 2023-11-21

**Authors:** Bo Chen, Siting Huang, Liyang Zhang, Liting Yang, Yuanyuan Liu, Chuntao Li

**Affiliations:** ^1^ Department of Neurosurgery, Xiangya Hospital, Central South University, Changsha, Hunan, China; ^2^ Department of Surgery, LKS Faculty of Medicine, School of Clinical Medicine, Queen Mary Hospital, The University of Hong Kong, Pokfulam, Hong Kong SAR, China; ^3^ National Clinical Research Center for Geriatric Disorders, Xiangya Hospital, Central South University, Changsha, Hunan, China; ^4^ Department of Dermatology, Xiangya Hospital, Central South University, Changsha, Hunan, China; ^5^ Hypothalamic-Pituitary Research Center, Xiangya Hospital, Central South University, Changsha, Hunan, China

**Keywords:** intracranial aneurysm, hemodynamics, bibliometric, knowledge map, hotspot

## Abstract

**Background:** Hemodynamics plays a crucial role in the initiation, enlargement, and rupture of intracranial aneurysms (IAs). This bibliometric analysis aimed to map the knowledge network of IA hemodynamic research.

**Methods:** Studies on hemodynamics in IAs published from 1999 to 2022 were retrieved from the Web of Science Core Collection (WoSCC). The contributions of countries, institutions, authors, and journals were identified using VOSviewer, Scimago Graphica, and Microsoft Excel. Tendencies, frontier topics, and knowledge networks were analyzed and visualized using VOSviewer and CiteSpace.

**Results:** We identified 2,319 publications on hemodynamics in IAs. The annual number of publications exhibited an overall increasing trend. Among these, the United States, Japan, and China were the three major contributing countries. Capital Medical University, State University of New York (SUNY) Buffalo University, and George Mason University were the three most productive institutions. Meng H ranked first among authors regarding the number of articles and citations, while Cebral JR was first among co-cited authors. The *American Journal of Neuroradiology* was the top journal in terms of the number of publications, citations, and co-citations. In addition, the research topics can be divided into three clusters: hemodynamics itself, the relationship of hemodynamics with IA rupture, and the relationship of hemodynamics with IA treatment. The frontier directions included flow diverters, complications, morphology, prediction, recanalization, and four-dimensional flow magnetic resonance imaging (4D flow MRI).

**Conclusion:** This study drew a knowledge map of the top countries, institutions, authors, publications, and journals on IA hemodynamics over the past 2 decades. The current and future hotspots of IA hemodynamics mainly include hemodynamics itself (4D flow MRI), its relationship with IA rupture (morphology and prediction), and its relationship with IA treatment (flow diverters, complications, and recanalization).

## 1 Introduction

Intracranial aneurysm (IA) is a pathologically saccular or fusiform dilatation of the cerebral arteries that occurs in approximately 2%–5% of the population and can be life-threatening upon rupture (Brown and Broderick, 2014; Zhu et al., 2022). Because of the inevitable impingement of blood flow to the arterial wall, IA is closely related to hemodynamics. Once hemodynamic damage exceeds the structural strength of the arterial wall, the arterial wall is injured, and IA may occur. Hemodynamics interacts with other complex biological factors that contribute to IA initiation, development, growth, and potential stability or rupture (Frösen et al., 2012; Morel et al., 2021). However, in the early years, technical limitations made the measurement of hemodynamic parameters in humans difficult. The advent of computational and radiographic modeling has allowed for hemodynamic research on IAs. Studies have found that blood flow pulsation affects both the arterial wall surface (such as the wall shear stress and oscillatory shear index) (Soldozy et al., 2019) and inner structures (such as the medial gap and intimal pad) (Kataoka et al., 2020; Chen et al., 2022a), which can contribute to IA initiation, enlargement, and rupture. Hemodynamics also affects the biological signals of the arterial wall and can serve as a tool to understand the molecular pathogenesis of IAs (Levitt et al., 2019; Chen et al., 2022b; Chen et al., 2023). In addition, computational fluid dynamics (CFD) can be used to predict IA rupture (Tang et al., 2021), inform stent design (Suzuki et al., 2017; Bisighini et al., 2023), and allow surgical improvement (Bao et al., 2021). Overall, hemodynamic research in IAs is rich, diverse, and valuable. Clarifying the current status and hot topics may benefit new researchers in this field and permit better research.

Bibliometrics is a widely accepted method for reviewing numerous articles in a specific field through quantitative analysis (Donthu et al., 2021). Through bibliometrics, we can identify crucial contributors (e.g., authors, institutions, and countries), collaborative networks, and frontier research topics (Zhang et al., 2022). Several bibliometric studies on IAs have been conducted. Chen et al. (2022) investigated the research trends and hotspots of stent application in acutely ruptured IAs. Lu et al. (2021) described a research shift of unruptured IAs, especially in terms of endovascular treatment. Zhang et al. (2022) explored the application of animal models in IA research and found that mice were the optimal model (Chen et al., 2022). However, no bibliometric analyses of hemodynamic research in IAs have been reported to date. Accordingly, based on the Web of Science Core Collection (WoSCC) from 1999 to 2022, this study applied bibliometric tools (VOSviewer, CiteSpace, and Scimago Graphica) to uncover publication trends, influential contributors, top collaborators, and emerging frontier topics in the field of hemodynamic research in IAs.

## 2 Materials and methods

### 2.1 Data source and search strategy

The literature search was performed on the WoSCC website (https://www.webofscience.com/wos/woscc/advanced-search) to identify publications indexed between 1 January 1999, and 31 December 2022. The specific search formula was as follows: Topic (TS) = (“intracranial aneurysm*” OR “cerebral aneurysm*” OR “brain aneurysm*” OR “intracerebral aneurysm*” OR “cranial aneurysm*”) AND TS = (“hemodynamic*” OR “haemodynamic*” OR “computational fluid dynamic*” OR “CFD” OR “4D-Flow MRI” OR “optical imaging modalities” OR “particle image velocimetry” OR “PIV” OR “particle tracking velocimetry” OR “PTV” OR “shear stress” OR “flow velocity” OR “flow rate”). To avoid bias, two independent investigators (B Chen and LY Zhang) performed the literature search and filtering and a senior researcher (CT Li) resolved any discrepancies in findings between these investigators.

### 2.2 Inclusion and exclusion criteria

This analysis included original review articles on the hemodynamics of IAs indexed in the WoSCC database between 1 January 1999, and 31 December 2022. The exclusion criteria were 1) unpublished papers, 2) articles requiring manual research, and 3) articles written in languages other than English. Of the 2,815 publications initially identified, 496 were excluded, and 2,319 were finally included in the analyses.

### 2.3 Data extraction and bibliometric analysis

The extracted bibliometric parameters included journal names, publication times, titles, countries/regions, institutions, authors, keywords, references, and citations. Journal impact factors (IFs) were collected from the most recent Journal Citation Reports (2022). In addition, VOSviewer (version 1.6.18), CiteSpace (version 6.1 R6), Scimago Graphica (version 1.0.26), and Microsoft Excel 2019 were used to perform the bibliometric analysis and visualization. Microsoft Excel was used for the time and contribution analyses. Scimago Graphica was used for the country collaboration analysis. VOSviewer was used to visualize the institutional cooperation map, author cooperation map, author co-citation network, and keyword co-occurrence network. CiteSpace was used to visualize the keyword and reference burst figures and reference co-citation network.

## 3 Results

### 3.1 Overall characteristics

**FIGURE 1:**
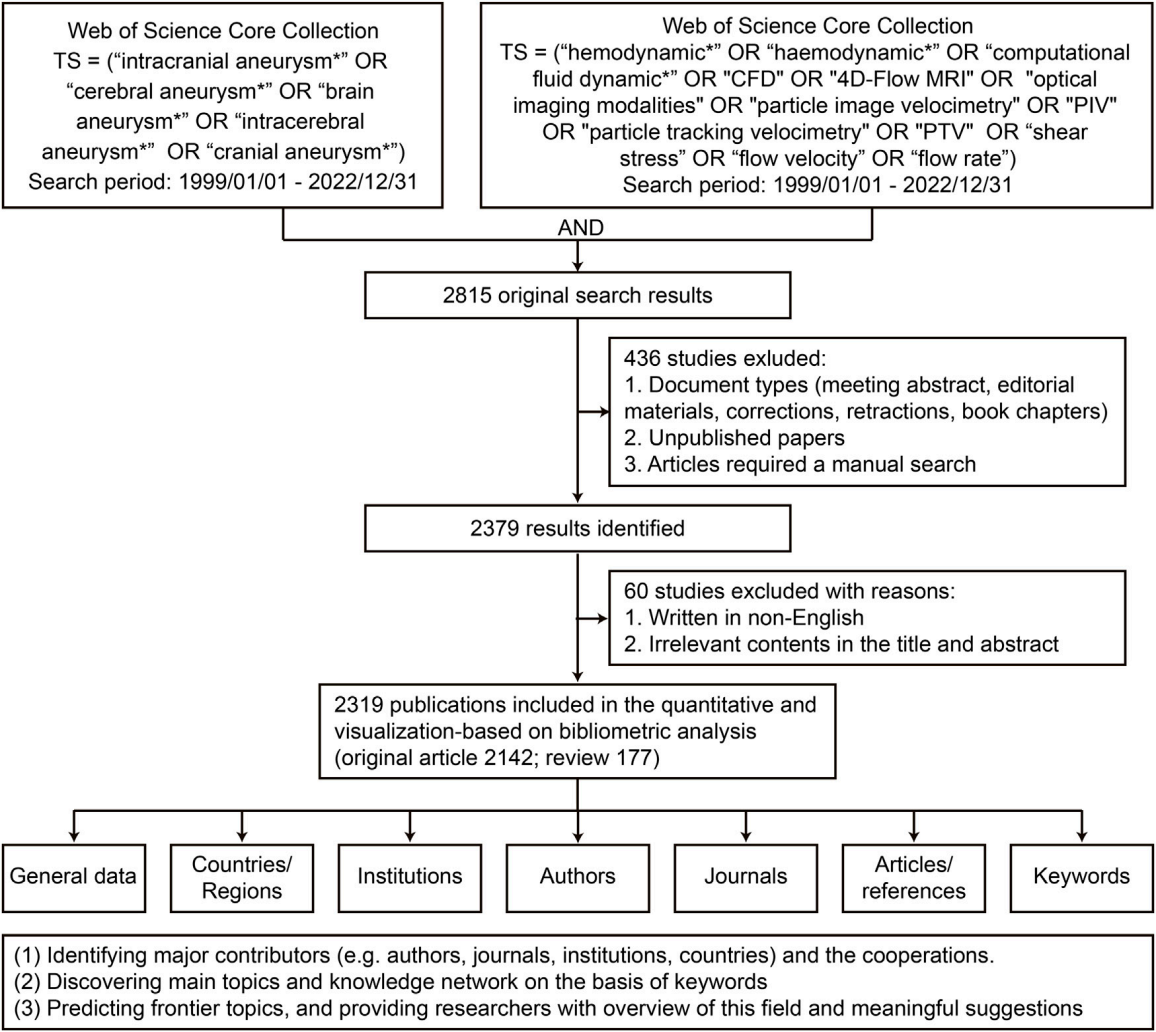
Flow chart of data collection, screening, and bibliometric analysis.

**FIGURE 2:**
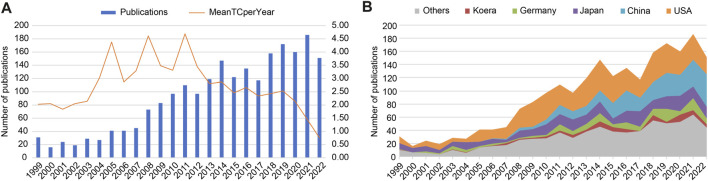
Trends of publications and citations on intracranial aneurysm (IA) hemodynamics. **(A)** Numbers of publications and mean total citations (TCs). **(B)** Annual publications of the top five countries/regions.

A total of 2,319 publications on the hemodynamics of IAs indexed between 1 January 1999, and 31 December 2022, were finally identified, including 2,142 (92.4%) original articles and 177 (7.6%) reviews (Figure 1). Although there were some slight fluctuations, the number of published articles showed an overall upward trend from 31 in 1999 to 151 in 2022, peaking at 186 in 2021. Additionally, the timing of the mean total citations (TC) per year could be divided into three periods: Phase I, 1999–2003 (remaining stable); Phase II, 2003–2013 (showing dramatic fluctuations); and Phase III, 2013–2022 (declining) (Figure 2A). Figure 2B shows the annual publications from the top five countries in this field. Among these, the US contributed the most publications. China began publishing articles in 2006, relatively late but has developed rapidly, surpassing the US in the number of published articles in 2021.

### 3.2 Countries/regions

**FIGURE 3:**
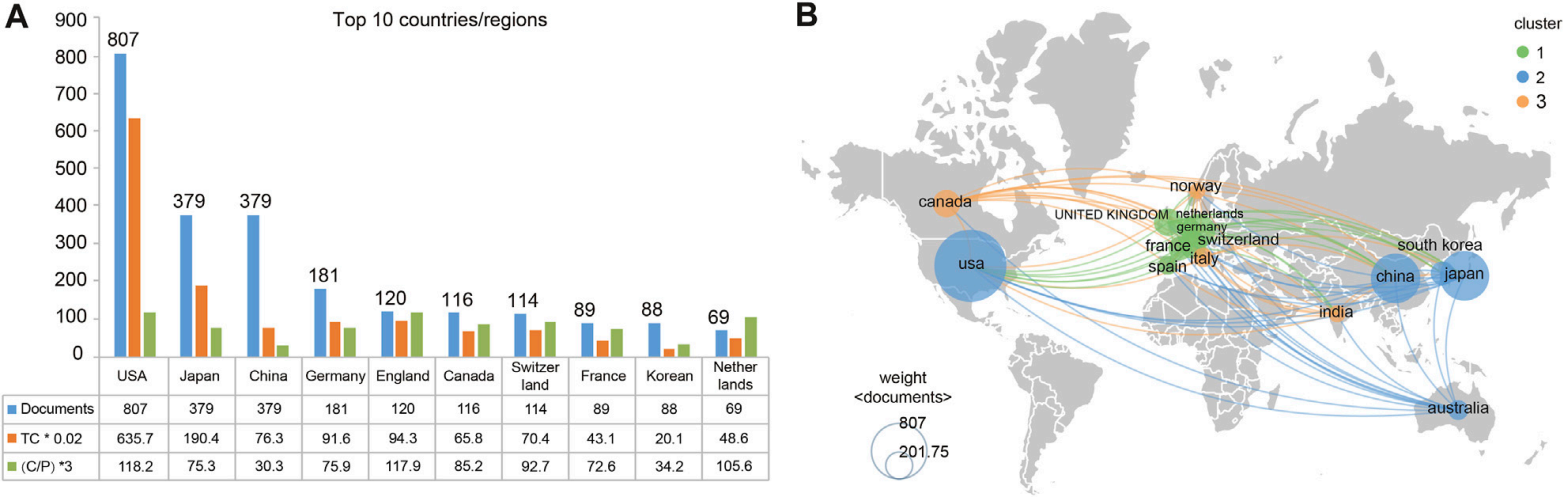
Top 10 most productive countries and international collaborations on IA hemodynamics. **(A)** Numbers of publications, TCs, and citations per paper (C/P). **(B)** International collaboration map. Node size, number of produced articles; color, clusters.

More than 60 countries/regions have contributed to this field, the top ten of which are listed in Figure 3. Among these, the US was first, with 807 publications, followed by Japan (379 publications) and China (379 publications). Regarding TC, the US again ranked first (31,785 TC), followed by Japan (9520 TC) and England (4716 TC). The US also ranked first in citations per paper (C/P) (39.4 C/P), followed by England (39.3 C/P) and the Netherlands (35.2 C/P) (Figure 3A). An international collaboration map drawn using VOSviewer with the minimum number of publications set at 40 included 15 countries/regions that met the criteria. Of these, the US, Japan, and China appeared as center nodes, with the closest cooperations between the US and Japan (link strength, LS = 69) and the US and China (LS = 52) (Figure 3B).

### 3.3 Institutions

**FIGURE 4:**
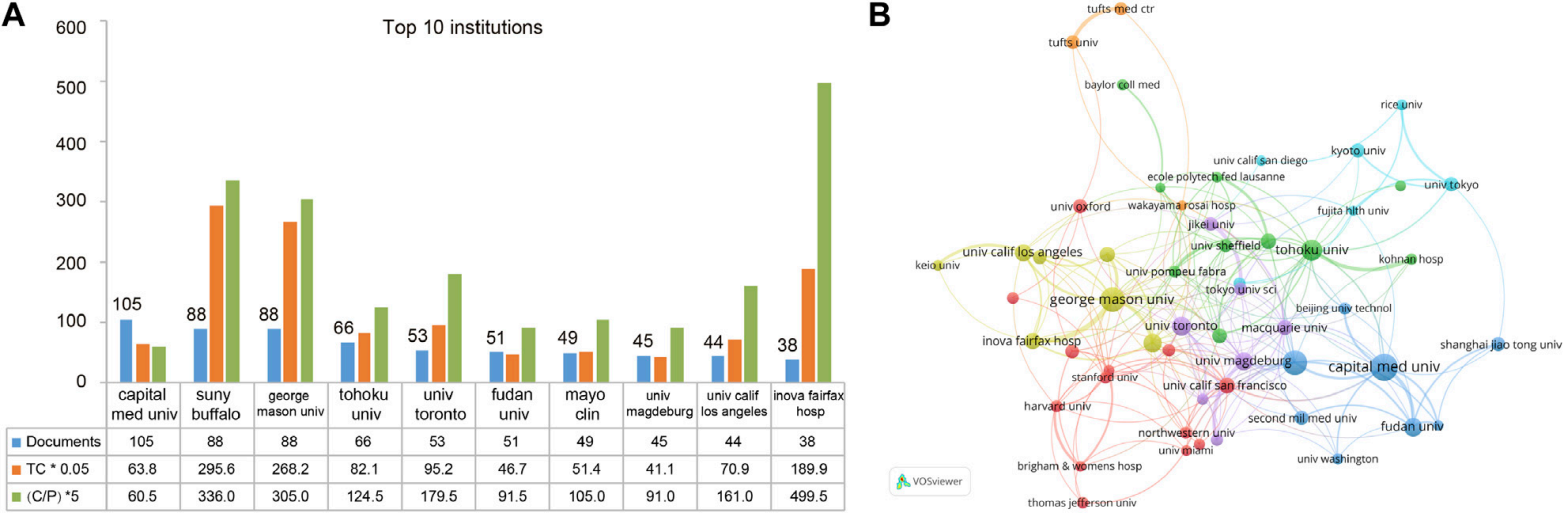
Top 10 most prolific institutions and inter-institution cooperations on IA hemodynamics. **(A)** Numbers of publications, TCs, and C/P. **(B)** Inter-institution cooperation network. Node size, number of produced articles; line thickness, cooperated strength; color, clusters.

In total, 861 institutions participated in the publication of articles on hemodynamics in IAs. Among the top ten productive institutions, five were located in the US, two in China, and one each in Canada, Germany, and Japan. Capital Medical University contributed the most publications (105 publications), followed by the State University of New York (SUNY) Buffalo (88 publications) and George Mason University (88 publications). Regarding TC and C/P, the top three institutions were SUNY Buffalo University (TC = 5,911, C/*p* = 67.2), George Mason University (TC = 5,364, C/*p* = 61), and Inova Fairfax Hospital (TC = 3,798, C/*p* = 99.9) (Figure 4A). Figure 4B illustrates the cooperation among the 54 institutions with >15 publications. Of these, George Mason University had the widest cooperation (total link strength, TLS = 108), followed by Capital Medical University (TLS = 60) and Tohoku University (TLS = 60) (Figure 4B).

### 3.4 Authors and co-authors

**FIGURE 5:**
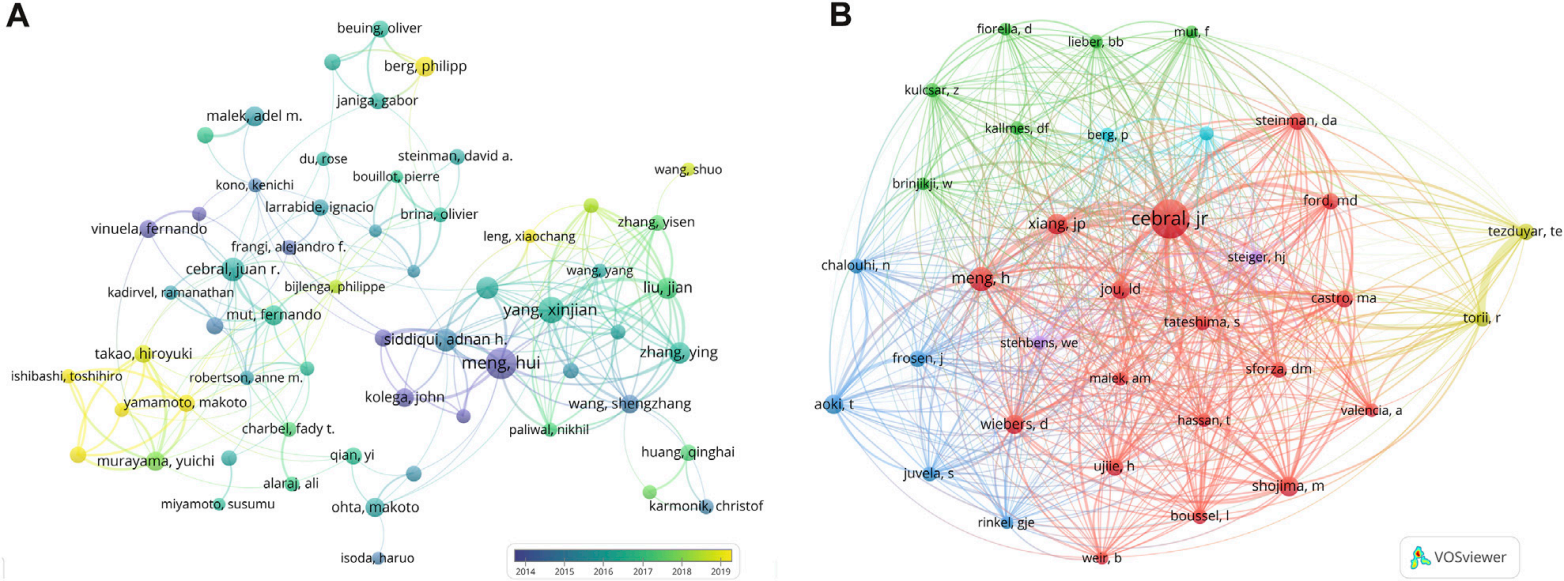
Author collaboration and co-cited author networks on IA hemodynamics. **(A)** Collaborated map of productive authors from 2014 to 2019. **(B)** Co-cited author map. Node size, number of produced articles; line thickness, cooperated strength; color, **(A)** average publication year, **(B)** clusters.

Table 1 shows the top ten prolific authors and the most co-cited authors, most of whom were from the US. Author co-citation was defined as ≥2 authors meanwhile cited in ≥1 publication. Among the authors, Meng, H published the most articles (69 articles, 4,326 citations), followed by Yang, Xj (50 articles, 929 citations) and Cebral, Jr (38 articles, 1,047 citations). The top three co-cited authors were Cebral, Jr (1902 co-citations), Meng, H (743 co-citations), and Xiang, Jp (532 co-citations). The visualized map analysis revealed that widely cooperating authors, including Meng, H (TLS = 173) and Yang Xj (TLS = 192), were active in the relatively early phase (average publication years 2013–2016), while recently active authors, such as Ishibashi, T and Berg, P (average publication year 2019) had relatively narrow cooperation networks (TLS _Ishibashi_ = 70, TLS _Berg_ = 30) (Figure 5A). The map of the top 34 co-cited authors with >200 co-citations showed the highest number of co-citations between Torii, R and Tezduyar, Te (LS = 2,443) (Figure 5B).

**TABLE 1:** Top 10 prolific authors and co-cited authors on hemodynamics research in IAs.

Rank	Author	Publications	Citations	Country	Co-cited author	Co-citations	Country
1	Meng, H	69	4,326	United States	Cebral, Jr	1,902	United States
2	Yang, Xj	50	929	China	Meng, H	743	United States
3	Cebral, Jr	38	1,047	United States	Xiang, Jp	532	China
4	Siddiqui, Ah	36	2,020	United States	Shojima, M	489	Japan
5	Xiang, Jp	34	1,332	China	Wiebers, D	455	United States
6	Zhang, Y	33	703	China	Aoki, T	453	Japan
7	Liu, J	32	491	China	Jou, Ld	417	United States
8	Berg, P	29	394	Germany	Ujiie, H	380	Japan
9	Mut, F	28	602	United States	Steinman, Da	368	Canada
10	Malek, Am	28	801	United States	Castro, Ma	358	United States

### 3.5 Journals, co-cited journals, and impact factors

Altogether, 461 journals published articles on hemodynamics research in IAs. The top ten active journals and co-cited journals are listed in Table 2. Journal co-citation was defined as ≥2 journals being cited simultaneously in ≥1 publications. In terms of publication quantity, the *American Journal of Neuroradiology* was first, with 155 publications, followed by *Neurosurgery* (94 publications) and *World Neurosurgery* (92 publications). Regarding citations and co-citations, the *American Journal of Neuroradiology* ranked first (7,906 citations, 8,108 co-citations), followed by *Stroke* 4,722 citations, 7,166 co-citations) and *Journal of Neurosurgery* (4,030 citations, 5,499 co-citations). In addition, among these ten journals and co-cited journals, the highest and lowest IFs were 8.3 (*Stroke*) and 1.7 (*Interventional Neuroradiology* and *J Biomech Eng-T Asme*), respectively.

**TABLE 2:** Top 10 prolific journals and co-cited journals on hemodynamics research in IAs.

Rank	Journal	Publications	Citations	IF	Co-cited journal	Co-citations	IF
1	*Am J Neuroradiol*	155	7,906	3.5	*Am J Neuroradiol*	8,108	3.5
2	*Neurosurgery*	94	3,877	4.8	*Stroke*	7,166	8.3
3	*World Neurosurg*	92	992	2.0	*J Neurosurg*	5,499	4.1
4	*J Neurosurg*	86	4,030	4.1	*Neurosurgery*	5,138	4.8
5	*J Neurointerv Surg*	83	1,519	4.8	*J Biomech*	2,341	2.4
6	*J Biomech*	78	1,975	2.4	*Ann Biomed Ang*	1,993	3.8
7	*Interv Neuroradiol*	53	254	1.7	*J Neurointerv Surg*	1,700	4.8
8	*Stroke*	47	4,722	8.3	*J Biomech Eng-T Asme*	1,539	1.7
9	*Ann Biomed Eng*	46	2,265	3.8	*Neuroradiology*	1,275	2.8
10	*Int J Numer Method Biomed Eng*	44	843	2.1	*Acta Neurochir*	1,174	2.4

IF, impact factor.

### 3.6 Keywords

**FIGURE 6:**
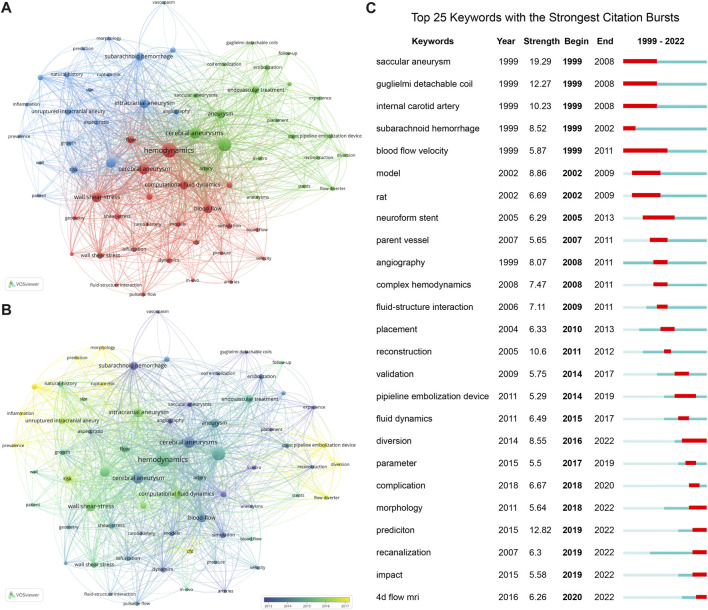
Analysis of keywords on IA hemodynamics. **(A)** Keyword co-occurrence network colored by clusters. **(B)** Keyword co-occurrence network colored by average publication years. Node size, keyword frequency. **(C)** Top 25 keywords with the strongest citation bursts. Red segment, burst duration.

Figure 6A illustrates a visualization of keywords that co-occurred at least 50 times in hemodynamics research in IAs. A total of 66 keywords were identified and grouped into three clusters. Cluster #1 (red) indicates research on hemodynamics itself, with keywords including “computational fluid dynamics,” “wall shear stress,” and “fluid-structure interaction”. Cluster #2 (blue) represents research on aneurysm rupture, with keywords including “arachnoid hemorrhage,” “rupture risk,” and “prediction”. Cluster 3 (green) indicates research on aneurysm treatment, with keywords including “endovascular treatment,” “stent pipeline,” and “coil embolization”. In addition, the keywords in Figure 6B are colored based on the average publication years. The concepts of “saccular aneurysms,” “carotid artery,” and “subarachnoid hemorrhage” appeared early (blue), while frontier topics including “inflammation” and “flow diverter,” appeared recently (yellow). Next, the CiteSpace burst module was applied to identify the research tendencies and shifts in central topics. Bursts refer to sudden increases over time. The 25 keywords with the strongest citation bursts are shown in Figure 6C. Among them, the topics gradually shifted from “saccular aneurysm,” “Guglielmi detachable coil,” and “internal carotid artery” to “angiography, complex hemodynamics, fluid-structure interaction” and “morphology, prediction, diversion, complication, recanalization, and four-dimensional flow magnetic resonance imaging (4D flow MRI)”.

### 3.7 Top cited articles and co-cited references

**FIGURE 7:**
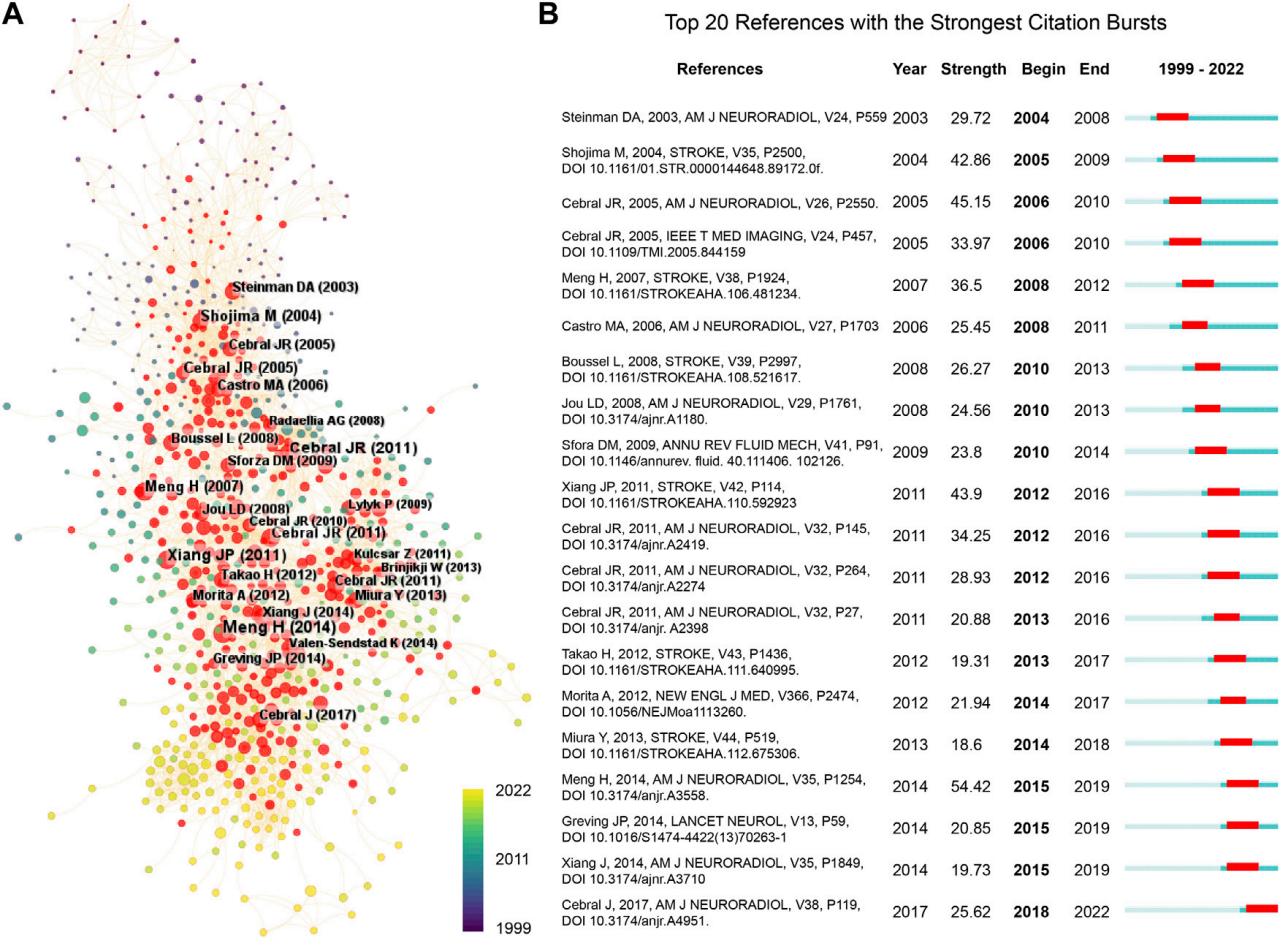
Analysis of co-cited reference on IA hemodynamics. **(A)** Reference co-citation network visualized using CiteSpace. Node size, citation number; red nodes, burst references. **(B)** Top 20 references with the strongest citation bursts. Red segment, burst duration.

Table 3 lists the top 10 most-cited papers in hemodynamics research on IAs, with the number of citations ranging from 351 to 566. Among these, nine were original articles and one was a review article. Four studies were published in *Stroke* and two in the *American Journal of Neuroradiology*. An article by Shojima et al. (2004) had the highest number of citations (566), followed by articles from Helgadottir et al. (2008) (550 citations) and Cebral et al. (2005) (514 citations). In addition, we performed a burst analysis of the reference co-citations. Reference co-citations are defined as two or more references cited in one or more papers simultaneously. Figure 7A shows the reference co-citation map colored by publication year from 1999 to 2022, in which the burst co-cited references were mainly concentrated in the middle period. Figure 7B displays the 20 references with the strongest citation bursts. Of them, “Meng et al., 2014, AM J NEURORADIOL, V35, P1254” had the highest burst strength (54.42), followed by “Cebral et al., 2005, AM J NEURORADIOL, V26, P2550” (strength = 45.15) and “Shojima et al., 2004, STROKE, V35, P2500” (strength = 42.86). The citation burst of “Cebral et al., 2017, AM J NEURORADIOL, V38, P119” ended in 2022, indicating high attention in recent years.

**TABLE 3:** Top 10 most cited publications related to hemodynamics research in intracranial aneurysms.

Rank	Title	Journal	Document type	Corresponding author	Affiliation	Year	Citations
1	Magnitude and role of wall shear stress on cerebral aneurysm: computational fluid dynamic study of 20 middle cerebral artery aneurysms	*Stroke*	Article	Kirino, T	University of Tokyo	2004	566
2	The same sequence variant on 9p21 associates with myocardial infarction, abdominal aortic aneurysm and intracranial aneurysm	*Nat Genet*	Article	Stefansson, K	deCODE Genet	2008	550
3	Characterization of cerebral aneurysms for assessing risk of rupture by using patient-specific computational hemodynamics models	*AJNR Am J Neuroradiol*	Article	Putman, CM	Inova Fairfax Hospital	2005	514
4	Hemodynamic-morphologic discriminants for intracranial aneurysm rupture	*Stroke*	Article	Meng, H	State University of New York (SUNY_ Buffalo	2011	481
5	High WSS or low WSS? Complex interactions of hemodynamics with intracranial aneurysm initiation, growth, and rupture: toward a unifying hypothesis	*AJNR Am J Neuroradiol*	Review	Siddiqui, A	SUNY Buffalo	2014	465
6	Efficient pipeline for image-based patient-specific analysis of cerebral aneurysm hemodynamics: technique and sensitivity	*IEEE Trans Med Imaging*	Article	Frangi, AF	George Mason University	2005	434
7	Complex hemodynamics at the apex of an arterial bifurcation induces vascular remodeling resembling cerebral aneurysm initiation	*Stroke*	Article	Kolega, J	SUNY Buffalo	2007	408
8	Prospective evaluation of surgical microscope-integrated intraoperative near-infrared indocyanine green video angiography during aneurysm surgery	*J Neurosurg*	Article	Spetzler, RF	Barrow Neurological Institute	2005	384
9	Aneurysm Growth Occurs at Region of Low Wall Shear stress patient-specific correlation of hemodynamics and growth in a longitudinal study	*Stroke*	Article	Saloner, D	Vet Adm Med Ctr	2008	366
10	Morphology parameters for intracranial aneurysm rupture risk assessment	*Neurosurgery*	Article	Kassell, NF	SUNY Buffalo	2008	351

## 4 Discussion

Hemodynamics is widely involved in IA formation, enlargement, and rupture (Soldozy et al., 2019; Liu et al., 2023). Hemodynamics can also be used to predict the prognosis and improve therapeutic approaches for patients with IA (Suzuki et al., 2017; Bao et al., 2021; Tang et al., 2021). In the current study, we performed a bibliometric analysis of publications on hemodynamics in IAs. This analysis identified publication trends, influential contributors (e.g., authors, journals, institutions, and countries), corresponding cooperation networks, and emerging frontier topics. Importantly, the results of this study may help new researchers to quickly learn about this field and perform better research in the future.

### 4.1 Overall trends and major contributing countries

The number of published articles reflects the interest of researchers in the field (Durieux and Gevenois, 2010; Huang et al., 2023). Overall, the number of publications on hemodynamics in IA displayed an upward tendency from 1999 to 2022, indicating the increasing interest of researchers in this field. Of these, the US was the most productive country, while China was an emerging country, with a steeper increase in the number of publications. Several factors may explain the increase in IA hemodynamics in China. First, China has a large population and a high prevalence of unruptured IA (7% among Chinese adults aged 35–75 years) (Li et al., 2013). Second, the number of neurosurgeons and the level of IA detection have grown strikingly in recent years. According to the reports of the World Federation of Neurosurgical Societies (WFNS), China has possessed the most neurosurgeons (around 11,000) worldwide since 2016 (Yu et al., 2019). Third, the Chinese government has recently expanded its funding in the field of basic research. However, an increased number of publications does not necessarily represent highly influential affiliations, authors, and articles. For example, China accounted for only two of the top ten productive institutions, one of the top prolific co-cited authors, and none of the top ten most cited articles.

The number of citations represents the performance of a publication (Durieux and Gevenois, 2010; Liang et al., 2023). In our study, the timing of the mean TCs on hemodynamics in IAs could be divided into three periods. Phase II (2003–2013) had a dramatically higher number of citations than the other two periods, reflecting better publication performance. During this Phase II, some representative keywords with high research value showed bursts, including “parent vessel,” “angiography,” and “fluid-structure interaction”. In addition, the top ten cited publications emerged during this Phase. Therefore, we conclude that Phase II was a crucial stage for research on hemodynamics in IAs, which laid the major foundation for current research. In addition, one reason for the declining citation phase of Phase III (2013–2022) might be the non-standardized acquisition of relevant hemodynamic parameters. Excessive assumptions, simplifications, and imprecise pre- and post-simulation steps may lead to incorrect findings in this field (Berg et al., 2019a) and could partially explain why hemodynamics have not yet been widely implemented for the investigation of IAs in clinical practice.

### 4.2 Active institutions, authors, journals, and co-cited journals

Identifying influential authors and institutions may help researchers choose their collaborating partners. Meng H (SUNY Buffalo University) was the most productive and cited author of hemodynamic research on IAs. She and her team performed CFD histology mapping on a dog IA model and found that high wall shear stress (WSS) and a high WSS gradient (WSSG) were dangerous hemodynamic conditions for IA initiation (Meng et al., 2007). Later, based on clinical imaging data, she built a combined model of hemodynamics and morphology to predict IA rupture (Xiang et al., 2011). These studies were the most cited publications and references with the strongest citation bursts, laying the foundation for subsequent research. Regarding prolific affiliations, Capital Medical University in China ranked first but ranked low in citations per paper. Several reasons may account for the contradiction between quantity and quality in China. First, the number of publications in China will surpass that in the US for the first time in 2021, indicating that Chinese publications might need more time to be cited. Second, compared to the US, a lower percentage of Chinese studies are published in high-IF journals such as *Stroke* (IF = 8.3).

Journal productivity represents the interest of a journal in a specific field; thus, the top co-cited journals can be regarded as authoritative journals. In research on hemodynamics in IA, the *American Journal of Neuroradiology*, *Neurosurgery*, and *World Neurosurgery* are the most productive journals, while the *American Journal of Neuroradiology*, *Stroke*, and *Journal of Neurosurgery* were the most frequently cited journals. If Chinese researchers want to improve their influence on IA hemodynamics, they should deepen their studies and select more influential target journals.

### 4.3 Research hotspots and frontier trends

Quick learning in a field can be obtained through keyword co-occurrence analysis (Ai et al., 2023). In our study, the major keywords could be divided into three clusters representing different topics and frontier trends.

Cluster 1 (red): Research on hemodynamics itself. Hemodynamic analyses in IA are mainly performed through approaches including CFD, 4D-Flow MRI, and optical imaging. CFD calculates the blood flow by solving the governing equations of fluid mechanics; therefore, the flow field in CFD is slightly virtual. 4D-Flow MRI measures blood flow *in vivo* and *in vitro*; thus, the flow field is more real (Kamada et al., 2022). Previous studies have reported a strong correlation between 4D flow MRI and CFD for the inflow hemodynamics of IA (Misaki et al., 2021). Despite being more consistent with the real hemodynamics in the human body, 4D flow MRI also has shortcomings, including relatively low spatiotemporal resolutions and limited accuracy due to imaging noise (Wu et al., 2022). Moreover, optical imaging techniques are commonly utilized for the *in vitro* hemodynamic validation of CFD and 4D flow MRI, as they offer well-controlled and high-resolution flow fields and do not require the use of ionizing radiation (Wu et al., 2022). The common optical imaging techniques research on hemodynamics in IA include particle image velocimetry (PIV), particle tracking velocimetry (PTV), and others (Liou et al., 2007; Medero et al., 2020). Some international studies on IA hemodynamics have reported that the accuracy of hemodynamic calculation is affected by model segmentation, boundary conditions, hemodynamic parameters, solver algorithms, and others (Steinman et al., 2013; Berg et al., 2015; Berg et al., 2018; Valen-Sendstad et al., 2018; Berg et al., 2019b; Voß et al., 2019). Researchers should combine actual clinical situations to evaluate IA hemodynamics and use optical imaging techniques to validate the results. Furthermore, to enhance the reliability of research on hemodynamics in IA, Berg et al. (2019a) proposed flow analysis standardization in comparison studies, as well as numerical investigations in uncertainty quantification and validation studies.

Cluster 2 (blue): Research on the relationship between hemodynamics and IA rupture. IA rupture comprises 80%–85% of non-traumatic subarachnoid hemorrhages and can lead to high mortality (Brown and Broderick, 2014). Considering the long-term impingement of blood flow on the arterial wall, IA rupture is closely related to hemodynamics such as WSS, WSSG, oscillatory shear index (OSI), flow patterns, and others (Soldozy et al., 2019). One meta-analysis identified average WSS as a protective hemodynamic parameter, whereas OSI and low shear index% (LSA%) were harmful hemodynamic parameters of IA rupture (Han et al., 2021). However, hemodynamics are complex and the role of WSS in IA rupture remains controversial. Zhang et al. (2018) found that an excessively high WSS in the parent artery could predict rupture of anterior communicating artery aneurysms. Accordingly, regarding the “high-versus-low WSS” controversy, Meng et al. (2014) proposed a widely accepted unifying hypothesis that low WSS and high OSI contributed to the rupture of large and atherosclerotic IA phenotypes, while high WSS and positive WSSG facilitated the rupture of small or secondary bleb IA phenotype. In addition, compared to unruptured IAs, ruptured IAs have more complex and unstable flow patterns (Byrne et al., 2014) such as a higher number of vortices (Xiang et al., 2011) and more complex inflow jet patterns (Futami et al., 2017). Some retrospective cohort studies have shown that hemodynamics can be integrated with geometric and clinically relevant information, such as IA site and focal wall enhancement, to predict IA rupture (Janiga et al., 2015a; Berg et al., 2019b; Larsen et al., 2020). The area under the curve (AUC) value for predicting IA rupture accuracy can reach 0.820–0.910 (Chen et al., 2020; Shi et al., 2021). However, prospective, large-sample, multicenter cohort studies are needed to compare hemodynamics and IA rupture.

Cluster 3 (green): Research on the relationship between hemodynamics and IA treatment. Over the past 30 years, multiple therapeutic approaches have been developed for IA, including clipping and endovascular treatment (coils, stents, and flow diverters). These treatments commonly change the hemodynamic status. Both coil embolization and flow diverters decrease intra-aneurysmal blood flow velocity and WSS, which explains their protection against rupture (Goubergrits et al., 2014; Jing et al., 2016). When treatments fail, the high WSS at the neck remnant could require recanalization, while a lack of decreased flow velocity and undiminished high-WSS areas might lead to postoperative rupture (Goubergrits et al., 2014; Chen et al., 2021). Moreover, hemodynamic research may be an effective tool to improve IA treatment. Janiga et al. (2015b) used CFD to identify an optimal flow-diverting stent for patient-specific IAs. Chen et al. (2021) employed CFD to propose the proximal densification of flow diverters to reduce IA rupture risk. The combination of CFD and structural analysis can optimize flow diverter design, including the weave angle and wire thickness (Suzuki et al., 2017). Notably, in our study, the keyword “diversion” appeared in the average time of 2017, indicating that the flow diverter received more attention in IA treatments. However, flow diverters still have limitations and complications such as in-stent stenosis (John et al., 2016), thromboembolic complications (Leung et al., 2012), and others. Future hemodynamic research on IA treatment should focus on these topics.

## 5 Limitations

This study had several limitations. First, we only collected literature from the WoSCC, which provided the most suitable data format for CiteSpace and VOSviewer. Other databases such as Scopus and PubMed were also used to confirm our findings. Second, our study only included publications written in English and excluded non-English publications, which may have caused a selection bias. Third, record updates in the WoSCC may have led to retrieval disparities.

## 6 Conclusion

In conclusion, this study drew a knowledge map of the top countries, institutions, authors, publications, and journals on IA hemodynamics over the past 2 decades. The current and future hotspots of IA hemodynamics mainly include hemodynamics itself (4D flow MRI), its relationship with IA rupture (morphology and prediction), and its relationship with IA treatment (flow diverters, complications, and recanalization).

## Data Availability

The original contributions presented in the study are included in the article/Supplementary material, further inquiries can be directed to the corresponding authors.

## Abbreviations

AUC, Area under the curve; C/P, Citations/papers; CFD, Computational fluid dynamics; FSI, Fluid-structure interaction; 4D flow MRI, four-dimensional flow magnetic resonance imaging; IF, Impact factor; IA, Intracranial aneurysms; LS, link strength; LSA%, Low shear index%; N, Number; OSI, Oscillatory shear index; PIV, Particle image velocimetry; PTV, Particle tracking velocimetry; TC, Total citation; TLS, Total link strength; WSS, Wall shear stress; WoSCC, Web of Science Core Collection; WFNS, World Federation of Neurosurgical Societies; WSSG, WSS gradient.
